# Specific Physical and Nutritional Preparation of a Professional Kata Karate Athlete: A Case Study with a Bronze Medallist from the Pan American Games

**DOI:** 10.3390/nu17020306

**Published:** 2025-01-16

**Authors:** José Manuel García-De Frutos, Daniel López-Plaza, Francisco Javier Martínez-Noguera, Manuel Sanz-Matesanz, Alejandro Martínez-Rodríguez, Luis Manuel Martínez-Aranda

**Affiliations:** 1 Facultad de Deporte, UCAM—Universidad Católica de Murcia, Guadalupe, 30107 Murcia, Spain; jmgarcia887@ucam.edu; 2 Faculty of Education, University of Zaragoza, 50009 Zaragoza, Spain; dlplazapal@unizar.es; 3 Centro de Investigación del Deporte de Alto Rendimiento, Universidad de Murcia, Campus de los Jerónimos, Guadalupe, 30107 Murcia, Spain; fjmartinez3@ucam.edu; 4 Faculty of Health Sciences, European University Miguel de Cervantes, 47012 Valladolid, Spain; msanzm@uemc.es; 5 Department of Analytical Chemistry, Nutrition and Food Science, Faculty of Sciences, University of Alicante, 03690 Alicante, Spain; amartinezrodriguez@ua.es; 6 Alicante Institute for Health and Biomedical Research (ISABIAL Foundation), 03010 Alicante, Spain; 7 Physical and Sports Performance Research Centre, Faculty of Sports Sciences, Pablo de Olavide University, 41013 Seville, Spain; 8 SEJ-680: Science-Based Training (SBT) Research Group, Faculty of Sports Sciences, Pablo de Olavide University, 41013 Seville, Spain

**Keywords:** karate, sport nutrition, athletic performance, body composition, strength gains

## Abstract

Background and objectives: In karate, particularly in the kata discipline, there is a notable lack of studies focused on specific physical preparation for competitions. This highlights an urgent need for more in-depth research into this crucial aspect of athletic training to optimize performance and athlete preparation. The objective of this study was to analyze the influence of a dietary plan combined with specific physical preparation on the performance and body composition of a professional kata athlete preparing for a Pan American championship. Methods: A 20-year-old elite female karateka (60.7 kg, 165.4 cm) followed a nutritional plan with an isocaloric diet. The strength and power of the upper and lower limbs were evaluated through countermovement jump (CMJ) and one-repetition maximum (1RM) tests in bench press and free squat over a five-month period before the competition. Results: Following the nutritional plan and physical preparation, the athlete’s body composition improved in terms of fat loss (from 12.17% to 10.68%) and increased muscle mass (from 51.45% to 53.09%). Moreover, these improvements translated into better performance in tests such as CMJ (from 38.29 cm to 44.14 cm), 1RM bench press (from 54.5 kg to 67.6 kg), and 1RM free squat (from 65.1 kg to 78.4 kg). Conclusions: This study demonstrates that a comprehensive approach to personalized physical, technical, and nutritional preparation over 16 weeks significantly improves muscle strength and performance in karate kata. The novelty of this intervention lies in the detailed description of the total workload, encompassing both physical and technical performance, with a specific plan tailored to the athlete’s needs. Additionally, the preparation was precisely designed for a specific tournament, addressing the sport’s unique demands.

## 1. Introduction

Currently, WKF karate competitions are organized into two disciplines, kata and kumite, which differ in terms of the athlete’s physical and physiological profiles as well as the characteristics of sports performance [1]. Kata athletes rely more heavily on anaerobic endurance with a lower contribution from aerobic endurance compared to kumite athletes [1,2].

In kata, key physiological aspects include cardiorespiratory endurance, muscle strength, and body composition, while physical characteristics comprise speed, stability, power, mobility, coordination, and agility [3].

Several studies in the literature have explored the physiological and physical distinctions between kata and kumite. These studies have primarily focused on evaluating metabolic consumption [1], stability [4,5], agility [6,7,8], and mobility and power [8], as well as the effect of specific kata and kumite techniques on parameters purely related to the physical performance of athletes [9].

Karate is a sport that combines repetitive patterns of strikes, varied stances, and sudden movements in a dynamic and competitive environment, requiring quick reactions and the effective use of both offensive and defensive techniques. [10]. It is a short-duration combat that demands maximum intensity and a high level of motor and functional skills such as speed, agility, muscular strength, flexibility, coordination, and balance [11,12,13].

Muscular strength, power, and agility are considered the most crucial elements of physical fitness in karate [14,15,16]. Athletes engage nearly all muscle groups during competition, though the two forms of the sport—kumite and kata—differ significantly in style and corresponding muscular demands. The kata discipline, meaning “form”, consists of a predetermined sequence of movements in various directions in space, executed with explosive speed against imaginary opponents [1].

Regarding metabolic consumption, Doria et al. [1] found a higher metabolic power demand in kumite athletes compared with kata athletes (an average of 155.8 mL/kg vs. 87.8 mL/kg), with a predominance of aerobic contribution. In kata, competition is characterized by an intermittent display of offensive and defensive karate techniques, representing real combat against fictitious opponents. Kata athletes, whether men or women, compete individually or in synchronized teams of three athletes [17]. Additionally, kata was originally created as a karate exercise to develop coordination and movement rhythm to enhance the karateka’s technique [18] and mental aspects such as concentration, focus, and discipline [19]. Athletes are evaluated on athletic and technical criteria. Athletic criteria include strength, speed, and balance, while technical criteria encompass stances (i.e., leg position), basic techniques, transitional movements, timing, correct breathing, concentration (“kime”), and adherence to the style [19].

To perform, athletes must execute one of the 102 katas from the WKF’s list [20]. Typically, the katas performed in competitions last between 90 and 180 s [21] and are chosen by athletes according to their experience, difficulty level, and the opponent’s skill [22]. During the preparation phase for a competition, training is one of the most critical components to achieve a successful outcome, along with complementary strategies for athletic health management (e.g., nutrition, physiotherapy, and psychological support) [23,24,25]. The intricate combination of training factors applied during the preparation process can significantly influence an athlete’s success in competition [25]. However, detailed information regarding these aspects in the kata discipline has yet to be described.

Given the importance of karate in recent years, it is necessary to study the effects of various training protocols in this sport. Athletes and coaches need to be aware of advanced and diverse training methods and their impact on athletic performance to achieve success. Implementing an effective training program is designed to optimally enhance an athlete’s strength, power, and motor performance [26,27].

In karate, specific training programs are diverse, particularly in their objectives and duration, with a noticeable emphasis on the kumite discipline compared to kata. For instance, Seelan and Subradeepan [28] analyzed the impact of functional strength training combined with karate skills training in kumite, involving 60 karatekas assigned to three groups: functional strength training, karate skills training, and a combination of both over 12 weeks. Similarly, Yazdani et al. [29] explored how group plyometric and resistance training influenced explosive power and maximum strength in 18 karatekas over a 9-week period, though without specifying the karate discipline. Expanding on this, Davaran et al. [30] evaluated the effects of an 8-week plyometric training program on 120 karatekas, who trained three times a week, again without clarifying the discipline. On the other hand, Margaritopoulos et al. [31] focused exclusively on kumite karatekas, studying 20 participants who trained 5–6 times per week (120 min per session). Their research compared unipedal and bipedal plyometric training methodologies, aiming to enhance lower limb explosive strength and correct asymmetries. These studies underline the diversity of approaches in karate training programs and the need for further research to address specific gaps, particularly in understanding the distinct requirements of kumite versus kata disciplines.

The aim of this case study was to evaluate the effect of a 16-week program combining strength training, HIIT, technical work, and personalized nutrition on the performance and body composition of a female karate kata athlete preparing for an international competition. It was hypothesized that this intervention, focused on increasing muscle strength, improving technique, and optimizing physical performance through a diet tailored to the preparation’s demands, would positively impact the athlete’s performance and body composition, thereby enhancing her performance in the 2019 Pan American Games competition.

## 2. Materials and Methods

### 2.1. Study Design

A case study was conducted to assess the impact of a strength and power training regimen on the performance and body composition of a professional female karateka. Baseline measurements were taken prior to the intervention, followed by a midpoint assessment halfway through, and a final assessment after five months.

The participant was informed of any potential risks and discomforts and completed a medical history questionnaire, subsequently signing a consent form. The study strictly adhered to the Declaration of Helsinki (Edinburgh revision, 2000) and the guidelines of the EU’s Good Clinical Practice (Document 111/3976/88, July 1990). Ethical approval was granted by the Ethics Committee of the University of Alicante (UA-2021-03-11).

### 2.2. Participant

The participant was a 20-year-old Venezuelan female karateka competing in the Kata discipline for the national team since age 5 with 15 years of experience, including 4 years at the professional level. She had been part of the Venezuelan national team for three years, training 15 h per week in technical skills and physical preparation, resting only on Sundays. Her primary focus was university studies and karate. She was in good health, medication-free, with no history of surgery and normal blood biochemistry. Potential changes in the body due to the female menstrual cycle were not considered, as the duration of the menstrual cycle, the length of its phases, and the timing of the luteinizing hormone peak vary greatly both between individuals and within individuals. Moreover, recent evidence indicates that the menstrual cycle phase does not affect women’s acute strength performance or their adaptations to resistance training [32].

### 2.3. Data Collection

#### 2.3.1. Body Composition

Body weight was measured at 9 a.m., under fasting conditions and wearing minimal clothing, using a Tanita BC-545n (Tanita Corporation, Arlington Heights, IL, USA) with a precision of 0.1 kg. Height in a standing position without shoes was measured with a Seca 213 stadiometer (Seca, Hamburg, Germany) with a precision of 0.1 cm. To minimize potential sources of variability in the bioimpedance analysis (BIA) system related to total body weight and height, a body composition assessment was conducted by a Level 3 anthropometrist following the recommendations of the International Society for the Advancement of Kinanthropometry (ISAK) [33]. All measurements were taken in the same location, the UCAM high-performance center, under ambient temperature and baseline conditions. The technical error of measurement was less than 1% for perimeters, circumferences, lengths, and heights and less than 5% for skinfolds.

Anthropometric measurements were taken following the ISAK II complete profile methodology [33]. Skinfolds, circumferences, lengths, and widths were measured using a caliper, flexible metal tape, a segmometer, and a pachymeter, respectively (Holtain, Crymych, UK). Bone and muscle mass were obtained using Rocha’s equation [34] and Lee’s formula [35], respectively. Fat mass was estimated using Carter’s formula [36], Faulkner’s formula [37], and Withers’ equation [38]. Residual mass was calculated by subtracting the sum of bone, muscle, and fat mass from total body weight. According to the Spanish Committee for Kinanthropometry, these methods are considered the most appropriate for high-performance athletes [39].

#### 2.3.2. Performance Measurements

(A) Lower body power test

In the countermovement jump (CMJ) test, participants performed a maximal vertical jump from a standing position without using an arm swing. Additionally, participants had to bend their knees to a 90° angle. To ensure proper CMJ execution, familiarization trials were conducted prior to the test session, and the protocol was standardized [40]. For CMJ measurement [41], a contact platform (Chronojump Boscosystem, Barcelona, Spain) was used, whose reliability and validity have been well-documented [42]. Flight time was measured to calculate jump height. Participants performed three trials with a 30 s recovery period between them. The best jump result was used for subsequent analysis [43].

(B) Strength test

The 1RM (one-repetition maximum) bench press and squat tests were conducted following the guidelines described by Schoenfeld et al. [44].

### 2.4. Nutritional Intervention Protocol

For the dietary/nutritional intervention, quantitative estimates of total energy expenditure were based on basal metabolism using the Harris–Benedict formula [45] and corrected body weight. The resting metabolic rate (RMR) was determined to be 1490 Kcal. Physical activity expenditure was estimated using standardized activity factors [46]. The proposed diet was based on an isocaloric intake designed to maintain habitual dietary patterns and nutritional distribution.

During the intervention period, the athlete had unlimited access to the dietitian to address any questions about the dietary programs. The software used to develop the diet plan was Dietopro.com (Valencia, Spain), a SaaS (Software as a Service) web application powered by cloud computing technology, which is accessed and run by the user directly from their web browser [47]. The macronutrients in the diet were distributed according to Table 1.

### 2.5. Specific Physical Training Protocol

All training sessions began with a 5 min elliptical warm-up at a power of 50 W. Weeks 1, 8, and 16 were used for physical control tests and body composition assessments, with week 1 being the initial measurement, week 8 the secondary measurement, and week 16 the final measurement. The rate of perceived exertion (RPE) was recorded immediately after each session. Each session was supervised by a physical trainer. Conditioning sessions were scheduled with a minimum interval of 48 h. The duration of technical work varied daily, ranging from 90 to 120 min with an RPE load of 6–7 [48]. For additional details regarding the practices and their periodization, refer to Table 2 and Table 3.

### 2.6. Statistical Analysis

Descriptive data and necessary statistical analyses were performed using Excel ToolPak (Microsoft Office 365, Redmon, Washington, DC, USA). SI units were used to ensure uniformity and standardization. Measurements were reported as absolute values (specific units), while variable progress was expressed as percentage changes. To visually represent the performance variables and changes across the study period, a radial chart was developed displaying multidimensional data. Results were interpreted on the basis of observed trends and practical significance.

## 3. Results

### 3.1. Body Composition

The karateka maintained a relatively stable weight throughout the study period, with an initial weight of 60.7 kg and a final weight of 61.3 kg, showing only a slight increase despite changes in body composition. This stability can be attributed to the athlete’s reported strict adherence to the nutritional plan, which was designed to provide 3000 kcal daily. The plan emphasized macronutrient distribution, including 3.9 g/kg/day of carbohydrates and 2.1 g/kg/day of protein, ensuring sufficient energy availability to support both intense physical training and recovery processes.

Table 4 presents the results of the body composition assessment, highlighting the changes observed between the beginning and the end of the intervention. While the athlete’s overall weight showed minimal variation, the data reveal significant improvements in body composition. Body fat percentage decreased, indicating a reduction in fat mass despite the slight increase in total weight. Measurable changes in body composition during the intervention were found, with evidence of muscle hypertrophy reflected in the increase in muscle mass from 51.45% to 53.09%, alongside a reduction in fat percentage highlighting the effectiveness of the intervention in eliciting favorable adaptations relevant to athletic performance.

### 3.2. Performance Results

The CMJ test showed a measurable improvement in the athlete’s explosive lower-body strength, with initial results of 38.3 cm increasing to 44.14 cm, representing a 15.28% enhancement in jump height, demonstrating the effectiveness of the training program in improving the athlete’s ability to generate rapid force, a key component of performance in karate.

The upper-body strength assessment also revealed substantial progress. The bench press one-repetition maximum (1RM) increased from 54.5 kg to 67.6 kg, reflecting a 24% improvement. Similarly, the squat 1RM rose from 65.1 kg to 78.4 kg, a 20.43% enhancement, showing notable gains in both upper- and lower-body strength, which are essential for the physical demands of the sport.

**Figure 1:**
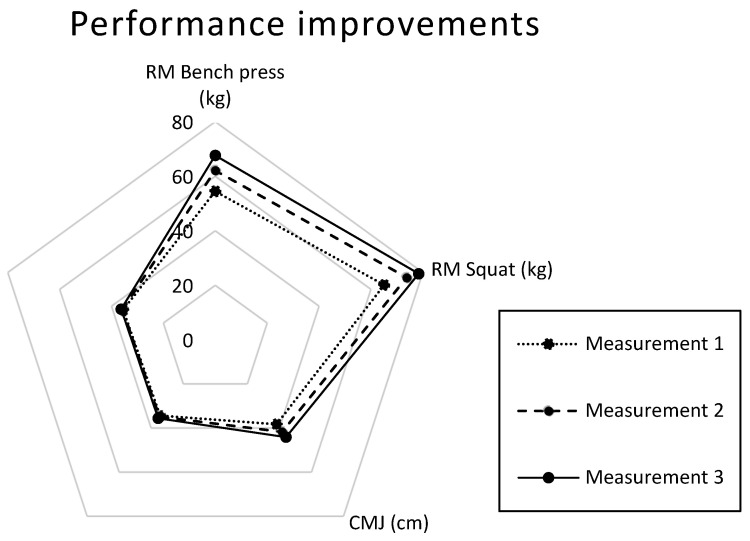
Physical performance results over the 5-month period in 1st, 2nd, and 3rd measurements; RM = repetition maximum.

Together, the improvements observed in the CMJ, bench press, and squat 1RM tests highlight the overall effectiveness of the intervention in enhancing strength and power across multiple muscle groups. For a visual representation of these results and their progression throughout the study period, refer to Figure 1.

## 4. Discussion

The purpose of this case study was to analyze the impact of a comprehensive 16-week program that included strength training, high-intensity interval training (HIIT) sessions, specific technical work, and a personalized nutritional strategy on the performance and body composition of a female karate kata athlete. The intervention combined key components of physical and technical training, along with nutrition tailored to the demands of preparation, aiming to enhance the athlete’s overall performance and specific readiness for an international competition.

This case study shares several relevant similarities with the study by Rossi [50], which help contextualize the results within the framework of high-performance female karate. Both investigations focus on female athletes of a similar age (20 years in the case study compared to a mean age of 21.2 years in Rossi’s study), with comparable body weight values (60.7 kg and 56.1 kg, respectively) and resting metabolic rate (RMR) (1490 Kcal versus 1448.6 Kcal).

These similarities indicate that female athletes in this discipline tend to maintain similar physiological parameters, suggesting a potential common physical profile among high-performance karate kata athletes. The slight differences in body weight and RMR could be attributed to individual variations in body composition, such as the ratio of lean-to fat mass, which directly affects basal metabolism.

The athlete’s body composition in this study showed higher fat mass and lower muscle mass at the beginning of the study compared with the end. This is also evident in the measurement of skinfolds, which decreased across all reference points except for the biceps and mid-thigh, which remained unchanged. Until now, no study has analyzed or reported these types of parameters in such depth in female karatekas.

In this line, another descriptive study on karate provided valuable data on the optimal percentage of body fat for this sport, ranging from 13–16% in young male athletes (27 ± 12.7 years) of different performance levels [51]. This range is slightly higher than the 10.68% body fat recorded by the karateka at the end of the intervention. Although differences in sex and age should be considered, this value may serve as a relevant benchmark for optimal body fat levels in female athletes practicing this sport.

Additionally, a study by Ojeda-Aravena et al. [52] on taekwondo athletes of both sexes (a combat sport with similar physical demands to karate) highlighted the correlation between body composition, percentage of fat mass (FM), and percentage muscle mass (MM) and performance in the CMJ. They emphasized its importance as they observed that a low percentage of FM could explain 75% of the performance change in the CMJ, while an adequate level of MM accounted for 71% of the performance improvement. A low percentage of FM followed by adequate MM was associated with specific physical performance in taekwondo [52]. In a study by Kabadayı et al. [53], a 10% improvement in the CMJ test was observed after an 8-week intervention involving 32 participants (17 women and 15 men) with an average of 4 years of karate experience, although the specific modality practiced was not specified. The intervention consisted of a karate-specific training program combined with core strength training. These findings support the idea that specific training over 8 weeks can lead to adaptations in jump power in karate practitioners. However, the main limitation of these data is the inclusion of both young men and women, making a direct comparison with the results of this case study, which focuses on a single female athlete, more challenging.

Other combat sports, such as MMA, have emphasized that maximal strength plays a crucial role and is essential for developing high-speed qualities [54,55]. Kostikiadis et al. [56] reported significant improvements in upper and lower body strength (16–20%), while this study observed a 24% increase in upper body strength and a 20% increase in lower body strength, supporting these findings. However, differences in sample size and timeframe should be considered. Additionally, the combination of HIIT-specific protocols with strength/power training led to the concurrent development of muscle power, as demonstrated by reduced time to complete a 2000 m rowing test and increased average power output.

Another study revealed that 16 weeks of training among university taekwondo athletes significantly increased maximal and explosive lower limb strength, showing a positive linear correlation between both capacities. It also demonstrated a strong correlation (r < 1) between the maximal strength of muscle groups involved in executing technical actions like kicks, turns, and movements and explosive strength, which improved as a result of the intervention [57].

Similarly, results consistent with those of García-Asencio et al. [58] confirm that a combined training system focused on strength development improves athletes’ jumping ability within 16 weeks. Sánchez-Sixto et al. [59] presented comparable findings, demonstrating that combining strength and plyometric training effectively enhances jumping performance in female basketball players.

These findings suggest that a combined training system with specific methods for its development enhances explosive strength as a crucial capacity for executing techniques like kicks, turns, and movements, which is highly associated with maximal strength. This necessitates its development within the methodological logic of planning. These results align with other significant studies that employed similar methodologies, reinforcing the validity of these approaches and their effectiveness in developing explosive strength [60,61].

In this case study, the main limitations include the difficulty of generalizing the results due to the individual characteristics of the athlete, the absence of a control group, and the physiological variability among athletes, which limits the representativeness of the findings. Although the 16-week intervention period is relevant, it may be insufficient to assess long-term effects. Additionally, other relevant variables such as endurance, agility, and flexibility, as well as key psychological factors in competitive performance, were not considered. These limitations highlight the need for future research with larger sample sizes, control groups, and extended follow-up periods.

## 5. Conclusions

The study demonstrates that a 16-week comprehensive training program, encompassing both physical and technical aspects, leads to significant improvements in maximum strength of both the upper and lower body, as well as in lower body power. A particularly relevant aspect is that after just 8 weeks of intervention, notable advancements in strength, power, and body composition were already observed. Furthermore, the novelty of this work lies in its integrated approach, which combines physical and technical preparation in a planned and specific manner aimed at competition.

The results reinforce the importance of designing personalized training programs that integrate both physical and technical development, particularly in high-level disciplines such as karate kata. This pioneering approach provides valuable insights for coaches and professionals, contributing to the advancement of scientific evidence on specific training methods.

A key aspect of this study is the relevance of implementing a comprehensive combined training program for performance and technique, with an appropriately adjusted nutritional strategy throughout the process, without the need for modifications, even when the total workload is progressively increased.

## Figures and Tables

**Table 1:** Macronutrient distribution during the 5-month training period.

Macronutrients	Intake (g/kg Body Weight)
Proteins	1.8–2
Carbohydrates	5–8
Lipids	1–1.2

Note: g = grams; kg = kilograms.

**Table 2:** Weekly training schedule for physical conditioning and technical training.

Monday	Tuesday	Wednesday	Thursday	Friday	Saturday	Sunday
Practice 1	Rest	Practice 2	Rest	Practice 3	Rest	Rest
Technical	Technical	Technical	Technical	Technical	Technical	Rest

**Table 3:** Combined training system program.

Sessions 1 and 3																			
Week		1	2	3	4	5	6	7	8		9	10	11	12	**13**	**14**	**15**	**16**	**17**
Stages		General									Specific						**Competition**		
Mesocycle		Introduction	Conditioning		Basic Stabilizer		Basic Developer				Developer			Preparatory Control			**Competitive**		
Work		Test	AA	AA	HYP	HYP	HYP	HYP	HYP-TEST		Fmax	Fmax	Fmax	P	**P**	**P**	**Compet**	**Compet**	**Test**
Exercise			Load																Rest
A	- Squat–lunge		3 × 8 × 70%		3 × 8 × 80%		3 × 8 × 85%				4 × 5 × 90%			5 × 3 × 60%			3 × 2 × 50%		Sets 3′
B	- Countermovement jump - Box jump		3 × 3		3 × 3		3 × 3				3 × 3			3 × 3			3 × 3		Sets 3′ Exercises 1′
A	- Press dumbbells - Bench press		3 × 8 × 70%		3 × 8 × 80%		3 × 8 × 85%				4 × 5 × 90%			5 × 3 × 60%			3 × 2 × 50%		Sets 3′ Exercises 1′
B	- Push up - Chest medicine ball throw		3 × 3 × 5 Kg		3 × 3 × 5 Kg		3 × 3 × 5 Kg				3 × 3 × 5 Kg			3 × 3 × 5 Kg			3 × 3 × 5 Kg		Sets 3′ Exercises 1′
A	- Deadlift dumbbells - Deadlift		3 × 8 × 70%		3 × 8 × 80%		3 × 8 × 85%				4 × 5 × 90%			5 × 3 × 60%			3 × 2 × 50%		Sets 3′ Exercises 1′
B	- Horizontal jump - Sled push		3 × 3		3 × 3		3 × 3				3 × 3			3 × 3			3 × 3		Sets 3′ Exercises 1′
RPE			12–14								10–12			10–12			10–12		
Total Time Per Session (min)			70								80			75			70		
Session 2																			
HIIT training with a density of 1:1, interval duration of 20–25 s, with an intensity of all-out FCmax. Metabolic exercises were performed, with self-loading. A quantity of 10 exercises with 3 series and 2 min rest between series [49].																			
RPE										17–19									
Total Time Per Session (min)										30									

Note: HIIT: high-intensity interval training; RPE: rating of perceived exertion (Borg 20 scale); AA, anatomic adaptation; HYP, hypertrophy; FMax, maximal force; P, power; Compet, competition.

**Table 4:** Body composition results during the 5 months of preparation in the 1st measurement, 2nd measurement, and 3rd measurement.

	1st Measurement	2nd Measurement	3rd Measurement	Total
	Value	Value (% Change)	Value (% Change)	% Final Change
Weight (kg)	60.7	59.9 (−1.32%)	61.3 (2.34%)	0.99%
Height (cm)	165.4	165.4 (0%)	165.4 (0%)	0%
SF Triceps (mm)	12.8	10.2 (−20.31%)	10.4 (1.96%)	−18.75%
SF Subscapular (mm)	6	6 (0%)	5.8 (−3.33%)	−3.33%
SF Biceps (mm)	3	2.6 (−13.33%)	3 (15.38%)	0%
SF Iliac crest (mm)	6.8	5 (−26.47%)	4.6 (−8%)	−32.35%
SF Supraespinal (mm)	4.8	4.4 (−8.33%)	3.8 (−13.64%)	−20.83%
SF Abdominal (mm)	5.6	5 (−10.71%)	4.8 (−4.00%)	−14.29%
SF Thigh (mm)	18	14 (−22.22%)	12 (−14.29%)	−33.33%
SF Calf (mm)	6	5.6 (−6.67%)	6 (7.14%)	0%
PR Relaxed arm (cm)	27.7	27.5 (−0.72%)	27.5 (0%)	−0.72%
PR Contracted arm (cm)	29.2	30 (2.74%)	29.6 (−1.33%)	1.37%
PR Waist (cm)	66.8	66.2 (−0.90%)	66.7 (0.76%)	−0.15%
PR Hip (cm)	99	98 (−1.01%)	99.5 (1.53%)	0.51%
PR Leg (cm)	34.8	34.9 (0.29%)	35.2 (0.86%)	1.15%
PR Thigh (cm)	56.3	55.5 (−1.42%)	55.8 (0.54%)	−0.89%
% Fat mass	12.17	11.02 (−9.45%)	10.68 (−3.09%)	−12.24%
% Muscle mass	51.45	52.39 (1.83%)	53.09 (1.34%)	3.19%

Note: kg = kilograms; cm = centimeters; mm = millimeters; PR = perimeter; SF = skinfold.

## Data Availability

The data presented in this study are available upon request from the corresponding author.

## References

[1] Doria C., Veicsteinas A., Limonta E., Maggioni M.A., Aschieri P., Eusebi F., Fanò G., Pietrangelo T. (2009). Energetics of karate (kata and kumite techniques) in top-level athletes. Eur. J. Appl. Physiol..

[2] Chaabène H., Franchini E., Sterkowicz S., Tabben M., Hachana Y., Chamari K. (2015). Physiological responses to karate specific activities. Sci. Sports.

[3] Gaweł E., Zwierzchowska A. (2024). The Acute and Long-Term Effects of Olympic Karate Kata Training on Structural and Functional Changes in the Body Posture of Polish National Team Athletes. Sports.

[4] Gauchard G.C., Lion A., Bento L., Perrin P.P., Ceyte H. (2018). Postural control in high-level kata and kumite karatekas. Mov. Sport Sci. Sci. Mot..

[5] Mirmoezzi M., Sadeghi H., Jafari M., Lotfi L. (2018). The Effect of Fatigue on the Static and Dynamic Balance in Karate Kata and Kumite Elite Men. J. Sport Biomech..

[6] Nedeljkovic A., Mudric M., Cuk I., Jovanovic S., Jaric S. (2017). Does specialization in karate affect reaction time in specific Karate kumite situations?. ISBS Proc. Arch..

[7] Syaquro A., Rusdiana A. (2017). Comparison of Whole-Body Reaction and Anticipation Reaction Time Between Kata and Kumite in Karate. IOP Conf. Ser. Mater. Sci. Eng..

[8] Koropanovski N., Berjan B., Bozic P., Pazin N., Sanader A., Jovanovic S., Jaric S. (2011). Anthropometric and physical performance profiles of elite karate kumite and kata competitors. J. Hum. Kinet..

[9] Molinaro L., Taborri J., Montecchiani M., Rossi S. (2020). Assessing the Effects of Kata and Kumite Techniques on Physical Performance in Elite Karatekas. Sensors.

[10] Lygouras D., Tsinakos A. (2024). The Use of Immersive Technologies in Karate Training: A Scoping Review. Multimodal Technol. Interact..

[11] Filingeri D., Bianco A., Zangla D., Paoli A., Palma A. (2012). Is karate effective in improving postural control?. Arch. Budo.

[12] Pal S. (2020). Preventive Methods for Karate Injuries—A Review. J. Clin. Diagn..

[13] Güler M., Gülmez İ., Yilmaz S., Ramazanoğlu N. (2017). The Evaluation of balance performance for elite male karate athletes after fatigue. Int. J. Sports Exer. Train Sci..

[14] Kraemer W.J., Mazzetti S.A., Nindl B.C., Gotshalk L.A., Volek J.S., Bush J.A., Hakkinen K. (2001). Effect of resistance training on women’s strength/power and occupational performances. Med. Sci. Sports Exerc..

[15] De Villarreal E.S., Kellis E., Kraemer W.J., Izquierdo M. (2009). Determining variables of plyometric training for improving vertical jump height performance: A meta-analysis. J. Strength Cond. Res..

[16] Katic R., Srhoj L., Pazanin R. (2005). Integration of coordination in the morphological-motor system in boys from 7 to 11 years old. Coll. Antropol..

[17] Paludo A.C., Lassalvia C., Mazhak I., Cacek J., da Silva D.F. (2023). “We missed the psychological support”: A case study about the preparation of the Brazilian bronze medal kata team for the 2019 Pan American Games. Front. Psychol..

[18] Nakayama M. (1968). Karate Kata Heian 4.

[19] Funakoshi G. (2014). Karatê-dô Kyohan: O Texto Mestre.

[20] World Karate Federation (2019). Sports rules and Regulations.

[21] Lassalvia C.E., Julio U.F., Franchini E. (2021). Effects of simulated kata competition on upper-and lower-body power tests performance. J. Asian Martial Arts.

[22] Augustovicova D., Argajova J., Saavedra-García M., Matabuena-Rodríguez M., Arriaza R. (2018). Top-level karate: Analysis of frequency and successfulness of katas in K1 Premiere League. Ido Mov. Cult. J. Martial Arts Anthrop..

[23] Issurin V.B. (2010). New horizons for the methodology and physiology of periodization of training. Sports Med..

[24] McGuigan M. (2017). Monitoring Training and Performance in Athletes.

[25] Bompa T.O., Buzzichelli C. (2019). Periodization: Theory and Methodology of Training.

[26] Fleck S.J. (1999). Periodized strength training: A critical review. J. Strength Cond. Res..

[27] Walker C. (2011). Effects of an Eight-Week Strength Intervention in Mixed Martial Arts Techniques.

[28] Seelan A.P., Subradeepan A. (2018). Effect of functional strength training and karate skill training on coordination ability of college level karate players. Int. J. Yogic Hum. Mov. Sports Sci..

[29] Yazdani S., Aminaei M., Amirseifadini M. (2017). Effects of plyometric and cluster resistance training on explosive power and maximum strength in karate players. Int. J. Appl. Exerc..

[30] Davaran M., Elmieh A., Arazi H. (2014). The effect of a combined Plyometric-Sprint Training program on strength, speed, power and agility of karate-ka male athletes. Res. J. Sport Sci..

[31] Margaritopoulos S., Theodorou A., Methenitis S., Zaras N., Donti O., Tsolakis C. (2015). The effect of plyometric exercises on repeated strength and power performance in elite karate athletes. J. Phys. Educ. Sport.

[32] Colenso-Semple L.M., D’Souza A.C., Elliott-Sale K.J., Phillips S.M. (2023). Current evidence shows no influence of women’s menstrual cycle phase on acute strength performance or adaptations to resistance exercise training. Front. Sports Act. Living.

[33] Esparza-Ros F., Vaquero-Cristóbal R., Marfell-Jones M. (2019). International Standards for Anthropometric Assessment—Full Profile.

[34] Rocha M.S.L. (1975). Peso ósseo do brasileiro de ambos os sexos de 17 a 25 años. Arq. Anatomía Antropol..

[35] Lee R.C., Wang Z., Heo M., Ross R., Janssen I., Heymsfield S.B. (2000). Total-body skeletal muscle mass: Development and cross-validation of anthropometric prediction models. Am. J. Clin. Nutr..

[36] Carter J.E.L. (1982). Body composition of Montreal Olympic athletes. Physical Structure of Olympic Athletes.

[37] Faulkner J.A. (1966). Physiology of swimming. Res. Q. Am. Health Phys. Educ. Recreat..

[38] Withers R.T., Craig N.P., Bourdon P.C., Norton K.I. (1987). Relative body fat and anthropometric prediction of body density of male athletes. Eur. J. Appl. Physiol. Occup. Physiol..

[39] Ramón J., Cruz A., Dolores M., Porta J. (2009). Protocolo de valoración de la composición corporal para el reconocimiento médico- deportivo. Documento de consenso del grupo español de cineantropometría (grec) de la federación española de medicina del deporte (femede). Versión 2010. Arch. Med. Deporte.

[40] García-Pinillos F., Ruiz-Ariza A., Moreno del Castillo R., Latorre-Román P.A. (2015). Impact of limited hamstring flexibility on vertical jump, kicking speed, sprint, and agility in young football players. J. Sports Sci..

[41] Bosco C., Luhtanen P., Komi P.V. (1983). A simple method for measurement of mechanical power in jumping. Eur. J. Appl. Physiol. Occup. Physiol..

[42] De Blas X. (2012). Proyecto Chronojump-Boscosystem. Herramienta Informática Libre para el Estudio Cinemático del Salto Vertical: Medición del Tiempo, Detección del Ángulo de flexión sin Marcadores y Elaboración de Tablas de Percentiles [Chronojump-Boscosystem Project. Free Tool to Study Kinematics Data on Vertical Jump: Time Measurement, Markerless Flexion Detección de ángulos y datos percentiles]. Ph.D. Thesis.

[43] Müller D.C., Izquierdo M., Boeno F.P., Aagaard P., Teodoro J.L., Grazioli R., Radaelli R., Bayer H., Neske R., Pinto R.S. (2020). Adaptations in mechanical muscle function, muscle morphology, and aerobic power to high-intensity endurance training combined with either traditional or power strength training in older adults: A randomized clinical trial. Eur. J. Appl. Physiol..

[44] Schoenfeld B.J., Contreras B., Vigotsky A.D., Peterson M. (2016). Differential Effects of Heavy Versus Moderate Loads on Measures of Strength and Hypertrophy in Resistance-Trained Men. J. Sports Sci. Med..

[45] Seagle H.M., Strain G.W., Makris A., Reeves R.S. (2009). Position of the American Dietetic Association: Weight management. J. Am. Diet. Assoc..

[46] Ainsworth B.E., Haskell W.L., Whitt M.C., Irwin M.L., Swartz A.M., Strath S.J., O’Brien W.L., Bassett D.R.J., Schmitz K.H., Emplaincourt P.O. (2000). Compendium of physical activities: An update of activity codes and MET intensities. Med. Sci. Sports Exerc..

[47] García C.G., Sebastià N., Blasco E., Soriano J.M. (2014). Dietopro.com: una nueva herramienta de gestión dietoterapéutica basada en la tecnología cloud computing. Nutr. Hosp..

[48] Borg G. (1998). Borg’s Perceived Exertion and Pain Scales.

[49] Martínez-Rodríguez A., García De Frutos J.M., Marcos-Pardo P.J., Orquín-Castrillón J. (2018). Frequency of High Intensity Circuit Training and Diet. Effects on Performance and Health in Active Adults: Randomized Controlled Trial. Arch. De Med. Del Deporte.

[50] Rossi L. (2021). Basal metabolic rate for high-performance female karate athletes. Nutr. Hosp..

[51] Zagorski D., Gikova M., Penov R. (2015). Relationship between kinematic characteristics and morphological parameters in shotokan karate athletes. Res. Kinesiol..

[52] Ojeda-Aravena A., Azocar-Gallardo J., Galle F., García-García J.M. (2020). Relación entre las características de la composición corporal y el rendimiento físico general y específico en competidores de taekwondo chilenos de nivel nacional de ambos sexos: Un estudio observacional. Rev. Esp. Nutr. Hum. Diet..

[53] Kabadayı M., Karadeniz S., Yılmaz A.K., Karaduman E., Bostancı Ö., Akyildiz Z., Clemente F.M., Silva A.F. (2022). Effects of Core Training in Physical Fitness of Youth Karate Athletes: A Controlled Study Design. Int. J. Environ. Res. Public Health.

[54] James L., Beckman E., Kelly V., Haff G. (2017). The neuromuscular qualities of higher-and lower-level mixed-martial-arts competitors. Int. J. Sports Physiol. Perform..

[55] James L.P., Haff G.G., Kelly V.G., Connick M., Hoffman B., Beckman E.M. (2018). The impact of strength level on adaptations to combined weightlifting, plyometric, and ballistic training. Scand. J. Med. Sci. Sports.

[56] Kostikiadis I.N., Methenitis S., Tsoukos A., Veligekas P., Terzis G., Bogdanis G.C. (2018). The effect of short-term sport-specific strength and conditioning training on physical fitness of well-trained mixed martial arts athletes. J. Sports Sci. Med..

[57] Pereira L.G., Torres A.F.R., Lavandero G.C., Morales P.A.R., Melendres M.E.L., Mora M.E.R. (2021). Evaluación de la factibilidad de un sistema de entrenamiento combinado en el desarrollo de la fuerza explosiva de los miembros inferiores de los taekwondocas. Retos.

[58] García-Asencio C., Sánchez-Moreno M., González-Badillo J.J. (2016). Entrenamiento combinado de fuerza y ejercicios de saltos, efectos sobre el rendimiento en el salto vertical en un grupo de alto nivel de jugadores de voleibol durante una temporada completa de competición. Retos.

[59] Sánchez-Sixto A., Floría P. (2017). Efecto del entrenamiento combinado de fuerza y pliometría en variables biomecánicas del salto vertical en jugadoras de baloncesto. Retos.

[60] Myong-Won S., Hyun-Chul J., Jong-Kook S., Hyun-Bae K. (2015). Effect of 8 weeks of pre-season training on body composition, physical fitness, anaerobic capacity, and isokinetic muscle strength in male and female collegiate taekwondo athletes. J. Exerc. Rehabil..

[61] Monks L., Seo M., Kim H., Jung H., Song J. (2017). High-intensity interval training and athletic performance in Taekwondo athletes. J. Sports Med. Phys. Fit..

